# Rhabdomyolysis presenting with severe hypokalemia in hypertensive patients: a case series

**DOI:** 10.1186/1756-0500-6-155

**Published:** 2013-04-17

**Authors:** Zhang Wen, Li Chuanwei, Zeng Chunyu, Huang Hui, Li Weimin

**Affiliations:** 1 Department of Respiratory, West China Hospital, Sichuan University, Chengdu, PR China; 2 Department of Cardiology, Daping Hospital, The Third Military Medical University, Chongqing, PR China; 3 Department of Endocrinology, West China Hospital, Sichuan University, Chengdu, PR China

## Abstract

**Background:**

Rhabdomyolysis presenting with severe hypokalemia as the first manifestation of primary hyperaldosteronism is extremely rare.

**Case presentation:**

Two middle-aged Chinese females were admitted to our emergency department for muscular weakness and limb pain, and both have the history of early onset hypertension. Laboratory test showed elevated creatinine phosphokinase (4, 907 and 8, 531 IU/L) and extremely low serum potassium (1.38 mmol/L and 1.98 mmol/L). Rhabdomyolysis and severe hypokalemia were established as first diagnosis. Hypokalemic rhabdomyolysis was confirmed after nervous system disorders, autoimmune diseases and trauma were excluded. Adrenal computerized tomography scan and postural stimulation test revealed aldosterone-producing adenomas. They both received laparoscopic adrenalectomy and were stable at the 2-year follow-up visit.

**Conclusion:**

The two cases remind physicians to bear in mind the risk of hypokalemia-induced rhabdomyolysis among patients with primary hyperaldosteronism.

## Background

Primary hyperaldosteronism (PHA) is a common cause of secondary hypertension. The main clinical manifestations are resistant hypertension and hypokalemia. Hypokalemia induced by PHA is a chronic process and most patients can tolerant the symptoms of malaise, muscular weakness and fatigability. But in some extreme conditions, PHA can induce excessive potassium excretion followed by rhabdomyolysis. Rhabdomyolysis presenting with severe hypokalemia as the first manifestation is rare in primary hyperaldosteronism. We report two cases of PHA diagnosed successfully.

## Case presentation

### Case 1

A 45-year-old Chinese female presented herself with fatigue and limb pain since 10 days in the emergency department. A slight pain in the right femoribus internus gradually aggravated in severity and extent during the following 10 days, and progressively got the upper limbs, back and neck involved in. She reported about a four-year history of arterial hypertension treated with nitrendipine and captopril. She denied any acute infections, trauma or intoxication. Physical examination revealed slightly elevated blood pressure (143/80 mmHg) and tenderness in the limb muscles. The laboratory examinations (Table 1) showed extremely low serum potassium (1.38 mmol/L) and elevated CPK (4, 907 IU/L). Electrocardiogram (ECG) showed Q-T interval elongation and abnormal U wave.

**Table 1:** Laboratory data on admission

		**Case 1**	**Case 2**
Urinalysis			
	pH	8.00	7.00
	glucose	-	-
	protein	-	-
	ketone	-	-
CBC			
	WBC	11.40 × 109/L	8.38 × 109/L
	RBC	4.71 × 1012/L	4.68 × 1012/L
	Hb	129 g/L	139 g/L
	Hct	0.39 L/L	0.39 L/L
	Plt	158 × 109/L	255 × 109/L
Blood Chemistry			
	Alb	47.1 g/L	37.4 g/L
	TBIL	17.0 umol/L	13.8 umol/L
	ALT	61 IU/L	46 IU/L
	AST	123 IU/L	115 IU/L
	GLU	6.56 mmol/L	5.33 mmol/L
	BUN	7.15 mmol/L	2.69 mmol/L
	Cre	92.7 umol/L	58.8 umol/L
	UA	297.5 umol/L	176.0 umol/L
	Myo	28.28 ng/ml	
	CHOL	4.56 mmol/L	4.00 mmol/L
	TG	1.63 mmol/L	2.94 mmol/L
	HDL-C	1.56 mmol/L	0.83 mmol/L
	CK	4907 IU/L	8531 IU/L
	LDH	477 IU/L	335 IU/L
	Na	142.1 mmol/L	146.0 mmol/L
	K	1.38 mmol/L	1.98 mmol/L
	Cl	98.7 mmol/L	97.8 mmol/L
Arterial Blood Gas Analysis on Room Air			
	pH	7.432	7.487
	pO2	72.0 mmHg	75.2 mmHg
	pCO2	37.4 mmHg	43.1 mmHg
	HCO3-	24.4 mmol/L	31.9 mmol/L
	BE	0.4 mmol/L	mmol/L

Based upon these clinical features, we established hypokalemia and rhabdomyolysis as first diagnosis. A series of laboratory examinations were performed for differential diagnosis of rhabdomyolysis. Biopsy results of right biceps brachii muscle revealed degenerated and necrotic muscle fibers with some inflammatory cells infiltrating the perimysium. The electromyogram was normal, which excluded nervous system disorders. Rhabdomyolysis induced by autoimmune diseases were excluded for negative results of autoimmune antibodies.

Hence, we supposed hypokalemia induces rhabdomyolysis. Tests for synchronous serum and urine potassium (First time: serum potassium 3.10 mmol/L, urine potassium 57.98 mmol/24 h; Second time: serum potassium 2.87 mmol/L, urine potassium 48.63 mmol/24 h) illustrated excessive potassium loss. Normal pH in arterial blood eliminated the suspect of renal tubule diseases.

High dose oral potassium supplementation was initiated. Pain and weakness were relieved and serum CPK levels normalized within one week. However, the serum potassium level remained low (3.00 mmol/L) despite potassium supplementation.

**Figure 1:**
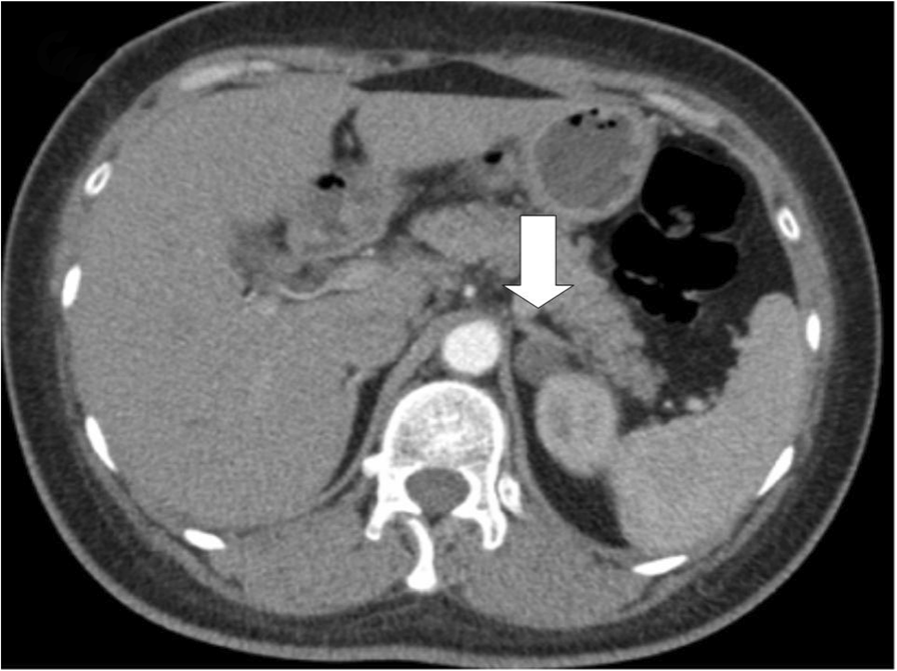
**Adrenal imaging of case 1.** Nodular mass (diameter approximately 21 mm) on the left adrenal gland, and the right adrenal gland appears normal.

**Figure 2:**
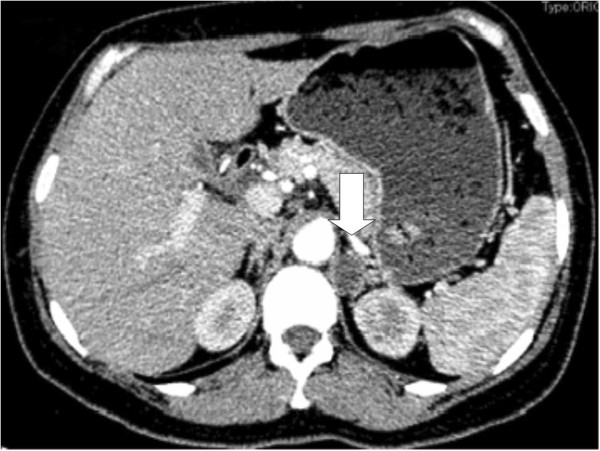
**Adrenal histology of case 1.** Fine connective tissue septa separate adrenal cortical clear cells nodules and the histopathology of resected specimen shows adenoma (HE × 100 and HE × 400).

Increased Aldosterone to Renin ration (ARR) indicated PHA (See Table 2 and Figure 1). Adrenal imaging with computerized tomography (CT) scan showed a low-density mass measuring 2.1 cm in diameter in the left adrenal (Figure 2). After initiation of spironolactone treatment, blood pressure and potassium levels in serum and urine returned to normal within one week.

**Table 2:** Endocrine test results

	**Case 1**	**Case 2**	**Reference range**	
Basal endocrine data: before postural stimulation test				
PRA	0.84	0.07	0.05-0.84	ng/ml.h
AT-II	28.61	43.63	28.2-52.2	ng/L
PAC	639.38	449.70	45-175	ng/L
ARR	76.12	642.43		ng/dl:ng
NE	<50	59	174-357	ng/L
E	<50	<25	60-104	ng/L
TSH	4.180	6.360	0.27-4.2	mU/L
T3	2.21		1.3-3.1	nmol/L
T4	129.30		62-164	nmol/L
FT3		4.42	3.60-7.50	pmol/L
FT4		17.59	12.0-22.0	pmol/L
Endocrine data: 2 hours after postural stimulation test				
PRA		0.17	0.56-2.79	ng/ml.h
AT-II		49.54	29.0-71.6	ng/L
PAC		265.80	98-275	ng/L

### Case 2

**Figure 3:**
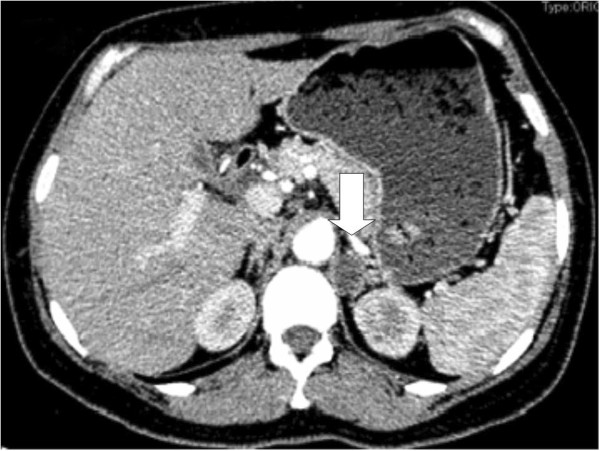
**Adrenal imaging of case 2.** Nodular mass (diameter approximately 16 mm) on the left adrenal gland.

Another Chinese female with 44 years old was admitted to our hospital with similar symptoms and signs of fatigue and limbs pain. Serum potassium was 1.98 mmol/L with highly elevated serum CPK levels (8, 531 IU/L). ECG showed highly elongated Q-T interval and abnormal apparent U wave. Differential work-up for hypokalemia and rhabdomyolysis showed PHA was the diagnosis (see Table 2 and Figure 1). CT scan revealed an adrenal gland mass with 1.6 cm in diameter (Figure 3). Symptoms were relieved after treatment with potassium and spironolactone.

**Figure 4:**
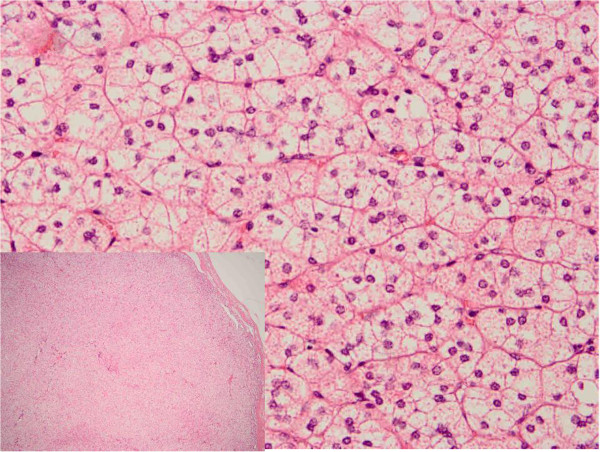
**Adrenal histology of case 2.** Adrenal cortical clear cells nodules is around by fine connective tissue septa and the histopathology of resected specimen shows adenoma (HE × 100 and HE × 400).

Both patients were treated with laparoscopic left adrenalectomy. Postural stimulation test and adrenal histology showed aldosterone-producing adenomas (Table 2 and Figure 4). Until the two year follow-up visit, the patients did not complain about similar symptoms anymore, and the blood pressures and potassium levels remained in a normal range.

## Discussion

Rhabdomyolysis is defined as a pathological condition of skeletal muscle cell damage leading to the release of toxic intracellular material into the blood circulation, such as CPK, myoglobin, aspartate aminotransferase, alanine aminotransferase and potassium [1-3]. The syndrome generally presents with the triad muscular pain, weakness and reddish brown urine [4].

The major causes of rhabdomyolysis include trauma, ischemia, drugs, toxins, metabolic disorders, infections and electrolyte disorders [5,6]. Especially, severe hypokalemia might play an important role in muscle cell damage [7,8]. Local potassium levels in capillaries are important regulators for vascular tension. Severe hypokalemia contracted capillaries, reduced muscle blood supply and finally resulting in lysing muscle cells and muscle cell damage [9,10]. Frank rhabdomyolysis usually occurs only when serum potassium values are below 2.0 mmol/L [8,11], which possibly induces cardiac arrhythmia and needs emergency treatment.

In PHA, aldosterone excess leads to water-sodium retention and potassium excretion. Patients usually present with hypertension and mild hypokalemia, while malaise and muscular weakness is always tolerable for patients. When vomiting or diarrhea occur or diuretics are used, serum potassium levels might drop to very low levels [12].

Although rhabdomyolysis usually results in hyperkalemia due to the direct release of intracellular potassium into the extracellular fluid, over-excreted aldosterone in PHA induces potassium excretion into urine. These mechanisms might be the reason we find hypokalemia, instead of hyperkalemia, resulting from rhabdomyolysis in those two cases.

## Conclusion

The diagnosis of PHA might be difficult when rhabdomyolysis and severe hypokalemia are the first manifestation. However, when rhabdomyolysis and hypokalemia occur in hypertensive patients, PHA should be considered. Further investigation for PHA should be initiated.

## Consent

Written informed consent was obtained from the patients for publication of this manuscript and accompanying images. A copy of the written consent is available for review by the Editor-in-Chief of this journal.

## Competing interests

The authors declare that they have no competing interests.

## Authors’ contributions

LW was primarily responsible for the conception, design and revision of the manuscript. ZW drafted the manuscript and searched the literature. ZC was responsible for manuscript editing and advice on literature review. HH was actively involved in the patients’ management and revised the manuscript. LC made substantial contributions to the acquisition of data. All authors read and approved the final manuscript.
